# Astrocytogenic bidirectional plasticity at spinal nociceptive synapses regulates acute nociceptive processing

**DOI:** 10.1097/j.pain.0000000000003805

**Published:** 2025-09-03

**Authors:** Sibel Ada, Laura Klinger, Hannah L. Teuchmann, Valeria Mussetto, Viktoria Hadschieff, Mira T. Kronschläger, Anna S.M. Siegert, Lidia Trofimova, Raphael Holzinger, Danijela Kurija, Ruth Drdla-Schutting

**Affiliations:** Division of Neurophysiology, Center for Brain Research, Medical University of Vienna, Wien, Austria

**Keywords:** Astrocyte, Spinal cord, Acute nociception, Plasticity, Gq-DREADDs

## Abstract

Astrocytes are key players in chronic pain, driving maladaptive changes in neuronal circuits. Yet, their influence on acute nociception—the body's first line of defense against harmful stimuli—remains poorly understood. Using chemogenetic tools to mimic endogenous astrocytic G-protein–coupled receptor-mediated signaling, we reveal that astrocytes induce bidirectional plasticity at nociceptive synapses in the dorsal horn. Depending on the state of these synapses and on concurrent afferent input, astrocytes can either suppress nociceptive transmission by enhancing glycinergic tone, or amplify synaptic strength at C-fiber synapses through interactions with microglia. This dynamic regulation expands the acute computational capacity of spinal nociceptive networks, enabling precise and adaptive responses to noxious stimuli on a short time scale. Our findings challenge the traditional view of astrocytes as passive supporters of neuronal activity and establish them as active regulators of spinal plasticity, integrating sensory information and network states to shape pain perception in real time.

## Abstract 2

Supplemental Digital Content is Available in the Text.

## 1. Introduction

Pain is the leading reason people seek medical care worldwide.^66^ Although commonly seen as a burden, acute pain serves an essential, life-preserving role by alerting organisms to actual or potential tissue damage and promoting protective behavior. To fulfill this critical function, nociception is tightly regulated through complex mechanisms that adapt pain perception to an organism's environment and physiological needs. Glial cells, and astrocytes in particular, have emerged as key contributors to this regulatory network, yet their role in acute pain remains poorly defined. Although astrocytes are often associated with maladaptive plasticity that exacerbates pathological, neuroinflammation-driven pain, our study suggests that in acute settings, astrocytogenic plasticity may play an adaptive role. By fine-tuning nociceptive signaling, astrocytes may help maintain pain at levels conducive to healing and homeostasis.

Astrocytes have emerged as active regulators in the pathogenesis of chronic pain. At the spinal level, changes in astrocyte morphology and upregulation of astrocytic markers are hallmark features of pathological pain, including neuropathic and chronic inflammatory pain.^33^ Pharmacological blockade of spinal astrocytes using glial toxins like fluoroacetate and fluorocitrate has been shown to alleviate or reverse established pain hypersensitivity in various models of pathological pain.^47,48,53,54,59^ Conversely, maintaining metabolic coupling between spinal astrocytes and neurons is essential for sustaining long-lasting pain states.^45^

Although these findings have advanced our understanding of spinal astrocyte involvement in pathological pain, much less is known about their role in acute nociceptive processing, particularly at the synaptic level. Astrocytes express a wide array of receptors, allowing them to rapidly sense and respond to both synaptically and non-synaptically released substances, facilitating the integration of synaptic inputs from diverse sources.^75^ Most of these receptors are G-protein–coupled receptors (GPCRs).^37,56^ Activation of these highly efficient signal transducers by even low levels of ligands promptly induces calcium signaling in astrocytes, regulating their functions.^1,3^ This signaling can trigger the release of neuroactive molecules, including gliotransmitters,^22^ which modulate neuronal activity and influence neighboring astrocytes and microglia on a short timescale.^15^ Indeed, several studies have shown that acute noxious stimuli can trigger coordinated actions among immune cells, immune-competent cells, vascular cells, and neurons within the spinal cord dorsal horn (SCDH),^25,35,42,70^ a phenomenon collectively termed neurogenic neuroinflammation.^77^

Here, we used Gq-coupled Designer Receptors Exclusively Activated by Designer Drugs (Gq-DREADDs) to actively manipulate intracellular calcium levels in astrocytes,^5,58^ allowing us to systematically investigate the downstream effects on acute nociception and pain through modulation of astrocytic excitability. Our findings revealed novel forms of astrocytogenic plasticity at the spinal level, demonstrating that spinal astrocytes can bidirectionally modulate nociceptive signals in response to concurrent peripheral input in acute settings.

## 2. Methods

Experiments were performed using male Sprague-Dawley rats, unless stated otherwise. These rats were either bred in-house at the Medical University of Vienna or sourced from Janvier Laboratories in Saint-Berthevin Cedex, France. The animals were maintained in standard conditions, with unrestricted access to both food and water, and they were subjected to a 12-hour light/dark cycle. All procedures were performed according to the ARRIVE guidelines and in accordance with the European Communities Council directives (86/609/EEC) and were approved by the Austrian Federal Ministry of Education, Science, and Research (BMBWF). To minimize bias, blinding strategies were implemented across experiments. In all major procedures, treatment group allocations of animals (DREADD vs Sham) were concealed from the experimenters during data acquisition and analysis, with unblinding occurring only after completion of data evaluation.

### 2.1. Spinal injection

Twenty-one to 25-day old rats were used for viral injections, which were performed as described elsewhere.^36,72^ Rats were anaesthetized with isoflurane (5 vol% for induction, 3.5 vol% for maintenance of anesthesia) and ventilated with a face mask. Respiratory rate resided between 80 and 90 bpm. The temperature was controlled via a rectal probe throughout and maintained at 35 to 37°C. Rats received eye ointment to prevent drying of eyes. Buprenorphine (0.025 mg·kg^−1^ s.c.) was used as analgesic. After the loss of tail-pinch reflex, a skin incision was made and the dorsal muscles dissected. A hemilaminectomy was performed to expose one side of the L4/L5 lumbar spinal cord. The spinal column was clamped in position using a clamp mounted onto a custom built frame. For injection, a borosilicate glass pipette with a tip opening of 30 to 40 µm was used. The tip of the needle was lowered to the spinal cord and positioned about 100 µm lateral to the central vein. The pipette was then lowered about 200 µm from the tissue surface. Approximately 500 nL of either *ssAAV-9/2-hGFAP-hM3D(Gq)_mCherry-WPRE-hGHp(A)* or *ssAAV-9/2-hGFAP-mCherry-WPRE-hGHp(A)*, both obtained from the Viral Vector Facility of the Neuroscience Center Zurich, Switzerland; titer 6 × 10^12^ vg/mL, was injected using a motorized microinjection pump (Nanoject III, Drummond Scientific Company) with an injection speed of 50 nL·min^−1^. A second injection was done spaced about 1 to 2 mm apart from the first one in rostrocaudal orientation. The injection glass pipette was left in place for additional 10 minutes after each injection to allow for diffusion of the injected compounds away from the pipette tip. After injection, the animals were released from the vertebral clamp, the isoflurane mixture was turned down to 2 vol%, the wound was sutured, and antibiotic ointment was applied. After awakening from anesthesia, rats were housed in single cages and monitored until they fully recovered. Carprofen (4 mg·kg^−1^ s.c.) was administered as a postoperative analgesic. One day after surgery, animals were reunited with littermates and housed in cages of 2 to 3.

### 2.2. Supraspinal injection

Projection neurons were retrogradely labeled as described in Ref. 31. Briefly, rats were deeply anesthetized with isoflurane (3.5 vol% for maintenance) and a ketamine/xylazine mixture (50 and 4 mg·kg^−1^, i.p., respectively). Rats were placed into a stereotactic apparatus and a hole was drilled (1.8 mm laterally and 0.5 mm caudally from the apex of the lambdoid suture) for insertion of a microsyringe at a 7° angle. Cholera Toxin Subunit B coupled with Alexa Fluor 555 (CTxB-555, 200 nL of a 1% solution) was infused at a rate of 0.1 µL·min^−1^ into the parabrachial nucleus. Carprofen (4 mg·kg^−1^, s.c.) was administered for postoperative analgesia. Only data gained from animals where the correct injection site was identified according to the atlas of Paxinos and Watson^55^ were used for further analysis. Robust expression of the fluorescent tracers in the spinal cord could be observed 3 to 5 days after the injection.

### 2.3. Slice preparation and in vitro electrophysiology

After a minimal waiting period of 10 days after spinal injection of an AAV—or 3 to 5 days after injection of a retrograde tracer—the animals were killed under deep isoflurane anesthesia, their spinal cords removed, and transverse spinal cord slices (around 500 µm thick) with dorsal roots attached were prepared. The slices were incubated for 30 minutes at 37°C and then stored at room temperature (∼21°C) in oxygenated incubation solution containing (in mM): 95 NaCl, 1.8 KCl, 1.2 KH_2_PO_4_, 0.5 CaCl_2_, 7 MgSO_4_, 26 NaHCO_3_, 15 glucose, 50 sucrose, pH was 7.4, measured osmolarity 310 to 320 mosmol·l^−1^. After transfer to the recording chamber, the slices were continuously superfused at a rate of 3 to 4 mL·min^−1^ with oxygenated recording solution. The recording solution was identical to the incubation solution except for (in mM): 127 NaCl, 2.4 CaCl_2_, 1.3 MgSO_4_, 0 sucrose. The recordings were conducted at room temperature. Virus expression was confirmed by visual inspection of the mCherry signal. Neurons located within lamina I and within an area of mCherry expression were visualized with Dodt infrared optics using a ×40, 0.80 N.A. water immersion objective on an Olympus BX50WI upright microscope.

Neurons were recorded in the whole-cell patch clamp configuration with glass pipettes (resistance = 2-4 MΩ) filled with internal solution (in mM): 120 potassium gluconate, 20 KCl, 2 MgCl_2_, 20 HEPES, 0.5 Na-GTP, 0.5 Na_4_-EGTA, 2 Na_2_-ATP, 7.5 phosphocreatine disodium salt hydrate, pH 7.4 was adjusted with KOH, measured osmolarity 310 mosmol·l^−1^. Biocytin (2 mg·ml^−1^, Biotium, #90055) was additionally included in the pipette solution. The resting membrane potential (mV) was measured after establishing the whole-cell configuration. Twenty 100-millisecond-long hyperpolarizing voltage steps from −70 to −80 mV were applied to each cell and averaged to calculate membrane capacitance (area under the capacitive transient), supplemental digital content (see Table S1, http://links.lww.com/PAIN/C383). For recordings of inhibitory postsynaptic currents (IPSCs), pipettes were filled with internal solution consisting of (in mM): 135 CsMeSO_3_, 5 TEA-Cl, 2 MgCL_2_,20 HEPES, 0.5 Na_4_-EGTA, 2 Na_2_-ATP, 0.5 Na-GTP, pH 7.4 was adjusted with CsOH, measured osmolarity 300 mosmol·l^−1^.

Spontaneous and evoked excitatory postsynaptic currents (EPSCs) were recorded at a holding potential of −70 mV, and for recordings of spontaneous and miniature IPSCs, the cells were clamped at a holding potential of 0 mV. In both cases, an Axopatch B700 patch clamp amplifier (Axon Instruments) and the pClamp10 acquisition software package was used. Spontaneous EPSCs were measured in the presence of bicuculline (10 µM), strychnine (1 µM), and D-2-amino-5-phosphonopentanoate (D-AP5, 50 µM) in the recording solution to isolate AMPAergic currents; spontaneous IPSCs were measured in the presence of 6-cyano-7-nitroquinoxaline-2,3-dione (CNQX, 10 µM) and D-AP5 (50 µM) at a holding potential of 0 mV. To isolate glycinergic IPSCs, bicuculline (10 µM) was added. In some recordings, TTX (1 µM) was added to record miniature IPSCs. Signals were low-pass filtered at 2 to 10 kHz, sampled at 20 kHz, and analyzed offline. No correction for the liquid junction potential was made. An image of the recorded neuron was taken after each experiment to measure the distance of the recorded neuron from the ventral border of the white matter using ImageJ software.

Excitatory postsynaptic currents were evoked by electrical stimulation of the dorsal roots using a suction electrode and recorded at a holding potential of −70 mV. Stimulation intensity was adjusted to activate C-fibers (1.8 mA on average), and conduction velocity was calculated by measuring the time from the stimulation artefact to the onset of the inward current. Fibers were classified as C-fibers when conduction velocity was below 0.5 m·s^−1^. Paired test pulses of 0.1 milliseconds were given at intervals of 15 milliseconds, to measure paired pulse ratio. An interstimulus interval of 50 milliseconds was used to ensure recovery to baseline levels before the second pulse.

The intensity of the test stimulation was set to twice the threshold for evoking C-fiber–meditated EPSCs. Monosynaptic EPSCs were identified by their constant latencies and absence of failures during repetitive stimulation of dorsal roots at 1 Hz. Excitatory postsynaptic currents that reliably followed a 10-Hz stimulation were classified as A-fiber–evoked and were not recorded further. Spontaneous (and miniature) EPSCs and IPSCs were recorded during 15-second traces over a time period of at least 15 minutes. Series resistance (<25 MΩ) was continuously monitored, and neurons were discarded when the series resistance changed by more than 30%.

Data were analyzed using GraphPad Prism 9 (GraphPad Software). Group sizes were calculated taking into account the type and number of planned comparisons, as well as the expected variability, based on previous experience and pilot data. Data are presented as mean ± standard error of the mean (SEM), and *P*-values <0.05 were considered statistically significant.

Synaptic strength was quantified by measuring the maximum peak amplitude of evoked EPCSs with Clampfit 10 software (Molecular Devices). Amplitudes of evoked EPSCs were normalized to the mean amplitude of the last 8 sweeps (2 minutes) before CNO application (“baseline” in the text). A one-way repeated measures ANOVA, followed by the Dunnett correction, or a paired *t* test was used to compare the different time points as indicated.

Spontaneous IPSCs and EPSCs were analyzed using Clampfit 10. Each trace underwent a preprocessing step where the initial 5 seconds were removed, leaving a 10-second segment for analysis. The data were then segmented into 5-minute bins for analysis, with the initial 5 minutes designated as the baseline. This was followed by the introduction of CNO during the wash-in phase, and subsequent removal of CNO during the wash-out phase. The full details of statistical tests for electrophysiological in vitro experiments can be found in supplemental digital content (see Table S1, http://links.lww.com/PAIN/C383). *P*-values refer to post-hoc comparisons, unless otherwise specified.

### 2.4. Animal surgery for in vivo electrophysiology

For the induction of anesthesia, an initial dose of isoflurane (4 vol% concentration) in a mixture of N_2_O/O_2_ (50:50) was administered within an induction chamber. Subsequently, the animals were intubated using a 16-G cannula and placed on mechanical ventilation at a rate of 75 strokes per minute, with a tidal volume ranging from 4 to 6 mL. To maintain anesthesia, 2 vol% isoflurane was used. The core body temperature of the animals was maintained at 37.5°C with the help of a feedback-controlled heating blanket. Adequate depth of anesthesia was confirmed by maintaining stable mean arterial blood pressure during noxious stimulation, which was measured using a physiological pressure monitoring device connected to the PowerLab 4/35 data acquisition system (AD Instruments).

The surgical procedures followed previous protocols.^39^ In brief, the animals had a jugular vein and a carotid artery cannulated for intravenous infusions and arterial blood pressure monitoring, respectively. Muscle relaxation was achieved through intravenous administration of pancuronium bromide. After cannulation, the left sciatic nerve was dissected free for bipolar electrical stimulation using a silver hook electrode. The lumbar segments L4 and L5 were exposed through laminectomy. The dura mater was delicately incised and moved aside. To immobilize the vertebral column, 2 metal clamps were used in a stereotactic frame. An agarose pool was created around the exposed spinal segments. A continuous superfusion of 5 mL of artificial cerebrospinal fluid consisting of the following composition (in mM): 135 NaCl, 1.7 KCl, 1.8 CaCl_2_, 10 HEPES, 1 MgCl_2_, pH 7.4 adjusted with KOH, with an osmolarity of 290 mosmol·l^−1^, was maintained using a roller pump within a closed circuit system. Additional drugs could be added to the superfusate as needed.

### 2.5. In vivo electrophysiology

In vivo electrophysiological recordings were performed as described previously.^39^ Briefly, C-fiber–evoked field potentials were recorded with glass electrodes (impedance 2-3 MΩ) from laminae I and II of the spinal cord dorsal horn in response to stimulation of sciatic nerve fibers. The pipette solution consisted of (in mM) 135 NaCl, 1.7 KCl, 1.8 CaCl_2_, 10 HEPES, 1 MgCl_2_, and 0.2% rhodamine B. At the end of each electrophysiological experiment, the recording site was labelled by pressure application (300 mbar for 1 minute) of 0.2% rhodamine B via the electrode. Electrodes were driven by a micro-stepping motor (Luigs & Neumann). Recordings were made with an extracellular amplifier (NPI electronic EXT-02 F) using a bandwidth filter of 0.1 to 1000 Hz. Signals were digitized and recorded using the PowerLab 4/35 acquisition system (AD Instruments). Afferent input from the hind paw was identified by mechanical stimulation of the foot while acoustically evaluating the evoked responses with an audio monitor. Test stimuli were delivered to the sciatic nerve and consisted of pulses of 0.5-millisecond duration at 25 V applied every 5 minutes using an electrical stimulator (ISO-01D-100, NPI Electronic). Conditioning low-frequency stimulation (LFS, 2 Hz, 2 minutes, 0.5 ms, 60 V) or low-intensity stimulation (50 Hz, 5 minutes, 0.1 ms, 100 mV) was also delivered via the sciatic nerve. Stimulation intensities were chosen based on previous publications.^11,17,79^

At the conclusion of each electrophysiological experiment, the animals were euthanized under deep anesthesia. The spinal cord was extracted and flash-frozen in isopentane for the detection of a rhodamine B spot at the recording site using a fluorescence microscope. Only experiments in which the recording site was situated in the superficial laminae and well within a region of high mCherry expression were included in the analysis.

For analysis, the area under the curve of C-fiber–evoked field potentials was determined offline using Clampfit 10. The mean area under the curve of 5 consecutive field potentials before CNO injection, or application of electrical nerve stimulation, respectively, served as a baseline. Responses were normalized to the baseline in every animal. Data were tested for normality using the Shapiro–Wilk test and for equal variances using Levene test. Statistical significance was tested with a paired t-tests, to compare the effects of treatments with baseline recordings. Details can be found in supplemental digital content (see Table S1, http://links.lww.com/PAIN/C383).

### 2.6. Electrophysiological recordings of astrocytes

Astrocytes were recorded in whole-cell patch-clamp configuration in transverse spinal cord slices obtained from either naive animals, or animals injected with either *ssAAV-9/2-hGFAP-hM3D(Gq)_mCherry-WPRE-hGHp(A)* or *ssAAV-9/2-hGFAP-mCherry-WPRE-hGHp(A)*, as described previously.^40^ Briefly, astrocytes were identified either by their characteristic morphology or by mCherry expression. Recordings were performed using glass pipettes (4-6 MΩ) filled with intracellular solution consisting of (in mM) 135 K-gluconate, 3 KCl, 10 HEPES, 1 EGTA, 0.3 Na_2_-ATP, 4 Mg-ATP, 0.1 CaCl_2_, 8 Na_2_-phosphocreatine, pH 7.28 adjusted with KOH, measured osmolarity 295 to 300 mosmol·l^−1^. The resting membrane potential was measured immediately after establishing whole-cell configuration. Current–voltage relationship (IV curves) were analyzed from 23 voltage steps of 10 mV step size each, starting from −160 to +60 mV.

### 2.7. Drugs for electrophysiology

The drugs were added to 20 or 40 mL of the recording solution to obtain the desired concentration as indicated below and then applied to the slices in a closed system by superfusion of the recording chamber. The DREADD ligand CNO (10 µM) and the AMPA/kainate receptor antagonist CNQX (10 µM) were diluted with DMSO, all other drugs were dissolved in water: the competitive NMDAR antagonist D-AP5 (50 µM), the GABA_A_ receptor antagonist bicuculline (10 µM), the glycine receptor antagonist strychnine (1 µM), and the voltage-gated sodium channel blocker TTX (1 µM); in some experiments, the GTP-analogue GDP-β-S (500 µM) or the calcium chelator BAPTA (20 mM) was added to the standard pipette solution.

For in vivo recordings, pancuronium bromide was given as an i.v. infusion (2 mg·kg^−1^·h^−1^). D-AP5 (50 µM) and minocycline (1 µM) were diluted in artificial cerebrospinal fluid (ACSF) and applied onto the spinal cord using a roller pump. CNO was administered as an i.v. bolus injection (3 mg·kg^−1^).

### 2.8. Behavior

#### 2.8.1. Mechanical and thermal withdrawal thresholds

Behavioral testing was performed during the light phase of the animals' light/dark cycle, in designated rooms (light intensity = 60-80 lux) and under constant testing conditions (temperature of 21°C ± 1°C, humidity of 55% ± 10%). Animals were habituated to the experimenter and the experimental setup on 2 days. Baseline mechanical paw withdrawal thresholds were assessed with von Frey filaments (Ugo Basile) on 2 consecutive days before the spinal injection of an AAV and averaged. Assessment of the mechanical withdrawal threshold was performed according to the simplified up-down method proposed by^4^ and described previously.^27^ Average values were calculated for the left and right hind paws. Thermal thresholds were assessed using the Hargreaves radiant heat test^28^ or the Heat Escape Threshold (HET) paradigm. In this paradigm, the temperature threshold required to elicit an escape response was determined using a punctate heat probe. An escape was defined as the withdrawal or movement of all 4 paws in response to stimulation of the hind paw. To determine the 50% HET, a modified version of the simplified up-down method (see above) was applied. Additional experimental details are provided in Ref. 30.

On the test day (day 0), the mechanical thresholds of the animals were reevaluated. Those animals whose thresholds were within the range of the baseline received an intraperitoneal (i.p.) injection of CNO (3 mg·kg^−1^). Animals whose values significantly deviated from the baseline were excluded from further experimentation. However, it is noteworthy that such instances were very rarely observed. Mechanical or thermal withdrawal or escape thresholds were assessed approximately 1.5 and 4.5 hours, and 24 and 48 hours after the CNO treatment. In some experiments, animals were anesthetized with isoflurane (4 vol%) delivered via a face mask to ensure complete immobilization. The animals were positioned on their backs, and care was taken to avoid any contact by the experimenter to minimize low-threshold, non-nociceptive input. CNO was administered i.p. through preimplanted cannulas. Isoflurane was discontinued 10 to 15 minutes later, and the animals were placed in individual cages to recover. Von Frey testing resumed approximately 1.5 hours after cessation of anesthesia, by which time all animals had fully recovered.

Behavioral data were analyzed using a two-way repeated measured ANOVA with Sidak post-hoc comparisons comparing treatments (DREADD vs Sham) and time points; details can be found in supplemental digital content (see Table S1, http://links.lww.com/PAIN/C383).

#### 2.8.2. Behavioral spectroscopy

To assess spontaneous behavior of freely moving animals, behavioral spectroscopy was used, as described previously.^7,16^ Briefly, the rats were put individually in the behavioral spectroscopy apparatus (Behavioral Instruments) without prior exposure to this setup for a total of 10 minutes, either immediately after the CNO injection or after the first assessment of either mechanical or thermal thresholds (around 90 minutes after CNO). The apparatus dimensions are 40 × 40 × 45 cm, and the light intensity within the apparatus was approximately 10 lux. During the experiment, animal behavior was recorded via video at a frame rate of 10-30 fps and analyzed in real-time using Viewer3 and the spectroscopy plug-in program (Biobserv). A customized classification software was used to detect various rat behaviors. The algorithm identified behaviors based on video, infrared, and vibration signals. The following behaviors were detected: rear (supported and unsupported standing on the hind paws), groom (behavior involving grooming of the head, face, flanks, abdomen, and back), still (motionless without head movements), time spent in locomotion, and scratch (rapid strong movements of legs). In addition, general track length (cm) and average locomotion velocity (cm/s) were assessed and calculated.

#### 2.8.3. Rotarod test

To investigate whether coordinated activation of spinal astrocytes influenced sensorimotor coordination and performance, a rotarod device (Ugo Basile, Italy) was used. Initially, a cohort of rats underwent habituation to the testing room followed by 2 consecutive days of training on the rod. During training, rats were placed on the rod set at 4 rpm over 60 seconds per trial. This training procedure comprised 3 trials separated by 10-minute intertrial intervals. Ideally, the rats were able to continuously walk on the rod, being placed back if they fell, although falls were rare. On the test day, rats received an intraperitoneal (i.p.) injection of CNO before being placed in separate lanes on the rod. The apparatus was set to accelerate from 4 to 40 rpm over 300 seconds, with a cutoff time of 500 seconds. This test procedure consisted of 3 trials separated by 10-minute intertrial intervals. The latency to fall (s) was recorded for subsequent data analysis.

### 2.9. Calcium imaging

Calcium imaging was performed using 500 µm thick transversal spinal cord slices, which were prepared as described above from animals that were injected with *ssAAV-9/2-hGFAP-hM3D(Gq)_mCherry-WPRE-hGHp(A)*. After cutting, slices were kept in the oxygenated incubation solution at 32°C for at least 30 minutes, after which they were stored in the same solution at room temperature. Acute slices were then transferred into the recording chamber and continuously superfused with oxygenated recording solution. After the verification of mCherry expression, the calcium indicator dye Oregon Green 488 BAPTA-1 AM was pressure injected into at least 3 different sites of the spinal cord dorsal horn. To do so, glass pipettes (resistance = 3-4 MΩ) filled with HEPES-buffered ACSF containing 353.3 μM OGB-BAPTA-1 AM dye, 1.6% Pluronic F-127, and 3.4% DMSO were used. After a 30- to 40-minute dye deesterification period, imaging was started at room temperature using a confocal microscope (DM6000CFS, Leica, Germany), equipped with a ×20 objective (Leica HCX APO, NA = 1.0). Time-series images were acquired at 1 Hz at a single focal plane. To assess functionality of the DREADD, CNO (10 µM) was bath applied for 5 minutes. To assess the calcium responses of dorsal horn cells in response to Aβ-fiber stimulation, the dorsal root was stimulated via a constant current stimulator in trains of 20 pulses at 20 Hz, at 25 µA and 0.1 ms pulse width,^62^ and imaging was continued for 60 seconds before, and after complete wash-out of CNO (10 µM), which was applied via the bath solution for 5 minutes, either alone or in combination with a 50-Hz continuous stimulation (50 Hz, 5 minutes, 25 µA, 0.1 ms).

Analyses of calcium imaging time-lapse image series were performed as described previously in 51, with slight modifications. Image series were analyzed offline using Fiji ImageJ (V1.54 t). BIO-Formats Plugins (release: 6.11.0) were used for Import and Data browsing of recorded time-lapse image series. XY-drift was corrected using a correction plugin (Multi DriftCorrection V1.1. by Brandon Brown). Recordings with a detectable Z-drift were excluded from further analysis. ROIs were defined for OGB-BAPTA-1-AM–positive cells and then classified as either mCherry^+^ (ie, astrocytes) or mCherry^−^ (ie, neurons) in laminae I, II, and III to IV. Time courses of fluorescence values for each ROI were analyzed using a bespoke program written in the Spike2 language (https://github.com/ronihogri/Calcium-signal-preprocessing-and-analysis-with-Spike2.git). Each data point was normalized as ΔF/F0 = (F(t)-F0)/F0. To correct for residual drift, F0 was defined locally using a sliding window encompassing the last 30 subthreshold data points, with the threshold set as the mean plus 2 standard deviations of the current window. The first 10 seconds of the recording (baseline period) were used as the starting window. Next, a high-pass filter (0.001 Hz, Butterworth) was applied. Calcium events were defined as periods in which ΔF/F0 values were at least 2 standard deviations higher than the baseline mean; the minimal event duration was set to 3 seconds. For each event, the peak amplitude was measured. The means of all mCherry^+^ and mCherry^−^ cells were then calculated for each slice. Peak amplitudes are shown for responders only. Statistical analysis was performed using one-way ANOVA, followed by Dunnett multiple-comparisons tests. Details can be found in the supplemental digital content (see Table S1, http://links.lww.com/PAIN/C383).

### 2.10. Histological analysis

#### 2.10.1. Characterization of Gq-coupled designer receptors exclusively activated by designer drugs expression

Animals were killed with sodium-pentobarbital (100 mg·kg^−1^ i.p.) 10 to 12 days after the injection of the viral vectors and transcardially perfused with 1% heparinized ice-cold saline solution, followed by 4% paraformaldehyde (PFA, 7.4 pH). The spinal cords were extracted and post fixed in PFA at 4°C overnight followed by cryoprotection in 20% and 30% sucrose in 0.1 M phosphate buffered saline (1 × PBS) for 24 hours each before being embedded in optimal cutting temperature compound (Sakura Finetek, Tokyo, Japan), flash-frozen in isopentane at −80°C, and stored at the same temperature. Transversal slices (40 µm thick) were cut using a cryostat and stored in well plates containing 0.05% Na-azide (Sigma Aldrich, S-2002) in 1 × PBS at 4°C until further processing. For in situ hybridization, PFA of 8.4 pH was used for perfusion; tissue slices were thaw-mounted onto cold object slides and stored at −80°C until further processing.

To perform free-floating indirect immunohistochemical (IHC) stainings, 40 µm tissue slices were washed in ×1 PBS + 0.1% Triton X-100 (Merck, 3 × 10 minutes) and then incubated for 60 minutes in a blocking solution of ×1 PBS + 0.1% Triton X-100 containing 4% normal goat serum (NGS, Cell Signaling Technology). All washing and incubation steps were performed on a shaker (70 rmp) at room temperature. Primary antibodies were added to the blocking solution. The next day, the slices were washed in ×1 PBS + 0.1% Triton X-100 (3 × 10 minutes) after which the secondary antibodies were applied in the blocking solution and incubated, protected from light, for 2 hours. Tissue slices were washed in ×1 PBS (3 × 10 minutes) and incubated in ×1 PBS + 0.2% 40,6-diamidino-2-phenylindole (DAPI, Abcam, ab228549), protected from light, for 60 minutes. Slices were again washed with ×1 PBS (2 × 5 minutes) and mounted on glass slides (Superfrost plus, Thermo Fisher Scientific) using Fluoromount-G mounting medium (Thermo Fisher Scientific). Glass slides were kept at 4°C at all times. Z-Stack images with 1 µm intervals were captured using an inverted confocal microscope (Leica TCS SP5, HCX PL APO CS), a ×40/1.25-0.75 oil immersion objective and the Leica application suite advanced fluorescence software (Leica Micro-system TCS SP5 LAS AF; Leica). Two spinal cord slices per animal were analyzed using IMARIS (Bitplane; V9.6.0).

#### 2.10.2. C-Fos expression in Gq-coupled designer receptors exclusively activated by designer drug animals

Animals were sacrificed 26 days after the injection of the viral vectors and 90 minutes post-CNO injection via transcardial perfusion. The spinal cords were then removed, postprocessed, sliced (40 µm), and stored as described above. Slices were stained using the free-floating indirect IHC staining protocol as described above. Images were captured with an inverted confocal microscope (Zeiss LSM780) using a ×20 dry objective and analyzed with IMARIS (Bitplane; V9.6.0).

#### 2.10.3. Characterization of biocytin-labeled cells

After in vitro electrophysiological recordings, all spinal cord slices were stored overnight in 4% PFA, pH 7.4, and then postprocessed in 20% and 30% sucrose in ×1 PBS for 24 hours each. Next, the slices were stored in 0.05% Na-azide in ×1 PBS at 4°C for free-floating IHC labeling. The detection of biocytin, Neurokinin-1 (NK1), and *Glycine* Transporter 2 (GlyT2) was performed according to the protocols described above, except the following: Primary antibodies were incubated for 90 minutes at room temperature, followed by incubation over night at 4°C. The secondary antibodies were incubated for 4 hours at room temperature. Next, z-stacks images with 1 µm intervals were captured using an inverted confocal microscope (Zeiss LSM780) and a LD LCI ×40/1.2 glycerol immersion objective. Images analysis was performed with ZEN Software (ZEN 2.3 SP1, Zeiss).

The following primary antibodies were used: anti-C-Fos from rabbit (1:500, Thermo Fisher Scientific, MA515055) or from guinea pig (1:1000, Synaptic Systems, #226005), mouse anti-NeuN (1:600, Millipore, MAB377), rabbit anti-Pax2 (1:100, Abcam, ab150391), guinea pig anti-NK1 (1:500, Millipore, AB15810), and chicken anti-GlyT2 (1:1000, Synaptic Systems, #272006). All secondary antibodies were raised in goat by Thermo Fisher Scientific and used accordingly to the listed primary antibodies as following: anti-rabbit Alexa Fluor 488 (1:500, A11008), anti-guinea pig Alexa Fluor 488 (1:500, A11073), anti-mouse Alexa Fluor 647 (1:500, A11008), anti-rabbit Alexa Fluor 647 (1:500, A21245), anti-chicken Alexa Fluor 488 (1:1000, A11039), anti-guinea pig Alexa Fluor 647 (1:250, A21450). Biocytin was stained with streptavidin coupled with Brilliant Violet 421 (1:1000, BioLegend, #406410).

## 3. Results

### 3.1. Selective chemogenetic activation of astrocytes depresses strength at spinal cord dorsal horn C-fiber synapses

We used GFAP promoter-driven Gq-DREADDs to selectively modulate astrocytic calcium signaling. Although these powerful tools are widely used to regulate neuronal activity,^60^ their application to control astrocytes, particularly within the SCDH, has been less explored. To validate the specificity of DREADD expression under our experimental conditions, we injected *ssAAV-9/2-hGFAP-hM3D(Gq)_mCherry-WPRE-hGHp(A)* into the SCDH of rats. This resulted in robust expression of Gq-DREADDs in astrocytes, with minimal off-target expression in neurons (see Fig. S1A, B, supplemental digital content, http://links.lww.com/PAIN/C382). Importantly, DREADD expression did not compromise astrocyte health or alter baseline electrophysiological properties (see Fig. S1C-G, supplemental digital content, http://links.lww.com/PAIN/C382). Functional integration of the DREADDs into the astrocytic membrane was confirmed by a clozapine-N-oxide (CNO)-induced (10 µM) increase in intracellular calcium in mCherry^+^ astrocytes in acute spinal slices, a response absent in astrocytes expressing a control construct (*ssAAV-9/2-hGFAP-mCherry-WPRE-hGHp(A)*, hereafter referred to as *Sham* animals) (see Fig. S1F, G, supplemental digital content, http://links.lww.com/PAIN/C382).

To explore the impact of CNO-mediated activation of astrocytic DREADDs on synaptic strength in the SCDH, we first recorded spontaneous excitatory postsynaptic currents (sEPSCs) from neurons located in close vicinity to mCherry^+^ cells (ie, DREADD-expressing astrocytes) in acute spinal slices. After a baseline recording period, CNO (10 µM) was bath applied for 5 minutes. Although this did not affect the sEPSC event rate in neurons across all laminae, it selectively reduced sEPSC amplitude in neurons located in lamina I and lamina II outer (see Fig. S2B-G, supplemental digital content, http://links.lww.com/PAIN/C382; for details, see Table S1, supplemental digital content, http://links.lww.com/PAIN/C383).

**Figure 1.**
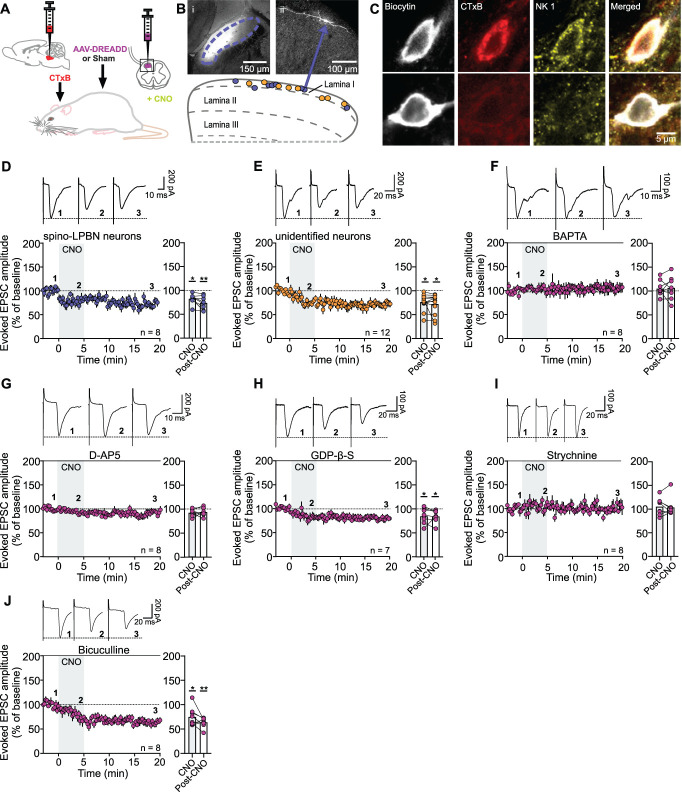
Chemogenetic activation of astrocytes induces *astroSD* at C-fiber synapses. (A) Diagram of AAV-DREADD injection into the SCDH and retrograde labeling of spino-LPBN. (B) (i) Typical CTxB injection site in the LPBN and (ii) representative biocytin-filled CTxB^+^ neuron. Scheme shows location of spino-LPBN (blue) and unidentified neurons (yellow). (C) Biocytin-filled recorded cells identified post-hoc: CTxB^+^ spino-LPBN cells expressed NK1R (top), whereas CTxB^−^ cells typically did not (bottom). (D–J) C-fiber–evoked EPSC amplitudes were normalized to baseline (BL, dashed line) and plotted against time. Bar graphs show BL-normalized means. CNO was bath applied for 5 minutes from time point zero (grey area). Insets show example traces from indicated time points. (D and E) CNO induced *astroSD* in spino-LPBN and unidentified neurons. (F) BAPTA and (G) D-AP5 prevented *astroSD*. (H) Blocking postsynaptic G proteins by GDP-β-S did not alter *astroSD* induction. (I) Strychnine-sensitive glycine receptor blockade abolished *astroSD*. (J) Blocking GABA_A_ receptors with bicuculline had no effect on the induction of *astroSD*. Statistics: One-way RM ANOVA, *P < 0.05; **P < 0.001. For details supplemental digital content (see Table S1, http://links.lww.com/PAIN/C383); data are shown as mean ± SEM.

Next, we investigated whether chemogenetic activation of astrocytes selectively affects synaptic subpopulations originating from primary afferent C-fibers terminating in the superficial laminae of the SCDH. Given that synaptic plasticity within the SCDH can be cell-type specific,^32^ we recorded evoked EPSCs from cholera toxin B (CTxB)-labeled projection neurons targeting the lateral parabrachial nucleus (LPBN). These neurons received monosynaptic C-fiber input and were confirmed to express the neurokinin-1 receptor (NK1R) by post-hoc immunostaining of biocytin-filled cells (Figs. 1A–C). Bath application of CNO induced a depression of synaptic strength at C-fiber synapses (Fig. 1D), a phenomenon we refer to as acute astrocytogenic synaptic depression (*astroSD*). Notably, *astroSD* followed a similar trajectory and magnitude in CTxB^−^ (i.e. unidentified) neurons from slices of the same animals (Fig. 1E), indicating that this effect was not restricted to the relatively small population of spino-LPBN neurons.

Further experiments (see Fig. S3, supplemental digital content, http://links.lww.com/PAIN/C382) revealed that *astroSD* was consistently induced in unidentified large-diameter neurons with a medio-lateral orientation in lamina I and monosynaptic C-fiber input in males (see Fig. S3A, B, supplemental digital content, http://links.lww.com/PAIN/C382), but not in females (see Fig. S3C, D, supplemental digital content, http://links.lww.com/PAIN/C382). Notably, aside from NK1R expression (Fig. 1C), no significant differences were observed between identified and unidentified neurons in terms of their location, input properties, cell capacitance, or membrane potential (see Table S1, supplemental digital content, http://links.lww.com/PAIN/C383). Therefore, for the remainder of this study, we focused on recordings from these neurons using male rats.

### 3.2. Signaling mechanisms of acute astrocytogenic synaptic depression

We next explored the mechanisms underlying this novel form of plasticity. Synaptic depression can involve pre- or postsynaptic mechanisms,^44^ yet the paired-pulse ratio—a measure linked to presynaptic release probability—remained unchanged after CNO (see Table S1, supplemental digital content, http://links.lww.com/PAIN/C383), indicating that *astroSD* relies on postsynaptic processes.

Elevations of calcium levels within neurons are crucial for synaptic depression at excitatory synapses.^84^ In line with these findings, *astroSD* was abolished when the calcium chelator BAPTA (20 mM) was included in the pipette solution (Fig. 1F), confirming its dependence on postsynaptic calcium.

Calcium-dependent reductions in synaptic strength have previously been associated with the activation of NMDA receptors (NMDARs)^29,43,78,80^ or metabotropic glutamate receptors.^23^ To investigate these pathways, we first applied the NMDAR antagonist D-AP5 (50 µM) to the bath, which blocked *astroSD* (Fig. 1G). In contrast, blocking postsynaptic G-protein signaling with the guanine nucleotide GDP-β-S (500 µM) did not impede the induction of *astroSD* (Fig. 1H), excluding the involvement of metabotropic receptors.

Next, we examined whether *astroSD* involved changes in inhibitory tone. CNO induced a significant increase in the rate—but not amplitude—of spontaneous inhibitory postsynaptic currents (sIPSCs) in the superficial SCDH of DREADD animals (see Fig. S4A, supplemental digital content, http://links.lww.com/PAIN/C382), in contrast to Sham animals (see Fig. S4B, supplemental digital content, http://links.lww.com/PAIN/C382). This effect was action potential-dependent, as it was absent in miniature IPSCs (mIPSCs) recorded in the presence of TTX (see Fig. S4C, supplemental digital content, http://links.lww.com/PAIN/C382). In line with these findings, administering bicuculline (10 µM) and strychnine (1 µM) via the bath solution to block GABA_A_ and glycine receptors while measuring C-fiber–evoked EPSCs prevented the induction, but not the maintenance of *astroSD* (see Fig. S4D, E, supplemental digital content, http://links.lww.com/PAIN/C382).

Given the predominance of glycine over GABA in modulating SCDH output under physiological conditions,^21^ we recorded glycinergic sIPSCs by bath application of bicuculline from lamina I neurons targeted by glycinergic terminals, which were identified by GlyT2 expression in post-hoc analysis of biocytin-filled cells (see Fig. S4F, supplemental digital content, http://links.lww.com/PAIN/C382). Post-CNO application, the rate of glycinergic sIPSCs significantly increased, whereas their amplitude remained unchanged (see Fig. S4G, Table S1, supplemental digital content, http://links.lww.com/PAIN/C382 and http://links.lww.com/PAIN/C383). Strikingly, blocking glycine receptors with strychnine while recording completely abolished *astroSD* (Fig. 1I), whereas GABA_A_ receptor blockade with bicuculline had no effect (Fig. 1J).

These findings suggest that *astroSD* at nociceptive synapses in the superficial SCDH is driven by postsynaptic calcium signaling and NMDAR activation, with glycine playing a central role. *Glycine*, released in an action potential–dependent manner, emerges as a key mediator of this novel form of plasticity, implicating glycinergic neurons as a probable source of this inhibitory transmitter.^21,81^

### 3.3. Stimulation of astrocytes induces mechanical hypersensitivity

**Figure 2.**
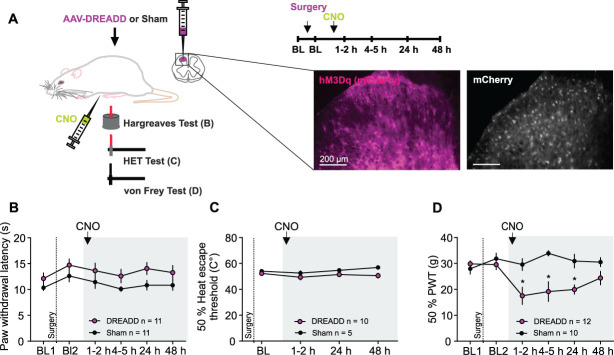
CNO induced mechanical hypersensitivity but had no effect on thermal thresholds. (A) Schematic of the experiment, with insets showing a part of the SCDH and mCherry expression (magenta or white). After establishing a baseline (BL), CNO was administered i.p. For further details, see Methods. (B) The paw withdrawal latency (s) was not affected by CNO. (C) The 50% heat escape thresholds (C°) remained stable over time. (D) The 50% mechanical paw withdrawal thresholds (PWT) were significantly reduced by CNO compared to BL in DREADD animals. In all cases, CNO had no effect in Sham animals (black circles). Statistics: Two-Way RM ANOVA, *P < 0.05; for details, supplemental digital content (see Table S1, http://links.lww.com/PAIN/C383). Data are shown as mean ± SEM.

Next, we investigated the impact of chemogenetic astrocyte activation on pain-related behavior in awake rats. We first measured thermal paw withdrawal latency at multiple time points after intraperitoneal (i.p.) injection of CNO (3 mg·kg^−1^; Fig. 2A). However, CNO had no effects on thermal thresholds, neither in the Hargreaves test (Fig. 2B) nor in the HET test paradigm, which is designed to assess heat aversion in rats^30^ (Fig. 2C), and did not impact motor function or exploratory behavior (Fig. S5, supplemental digital content, http://links.lww.com/PAIN/C382).

In contrast, CNO induced a significant decrease in paw withdrawal thresholds in response to stimulation with von Frey hairs. This mechanical hypersensitivity was transient and resolved approximately 48 hours later (Fig. 2D). CNO had no effect on thermal or mechanical thresholds in Sham animals (black circles in Figs. 2B–D).

### 3.4. Non-nociceptive input shifts the polarity of astrocytogenic plasticity

**Figure 3.**
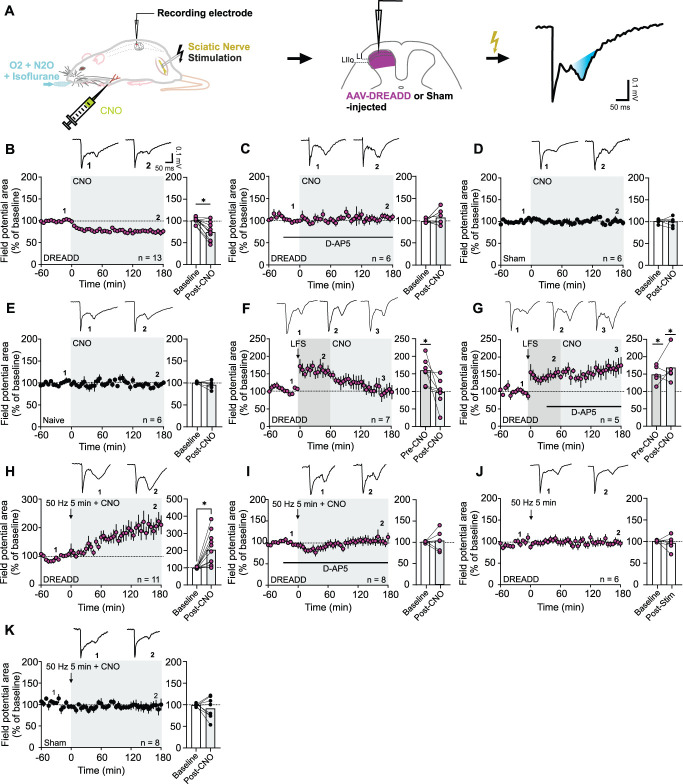
Bidirectional astrocytogenic plasticity at nociceptive synapses in vivo. (A) C-fiber–evoked field potentials recorded from the SCDH upon electrical sciatic nerve stimulation. (B-K) Field potential time courses (area under the curve) were normalized to BL (dashed line) and plotted over time (minutes). CNO was injected i.v. at time point zero in (B-E and H, I, and K), or at time point 60 in (F and G). Insets show representative original traces. Bar graphs show BL-normalized mean values for each time period. (B) CNO induced *astroSD* in DREADD animals. (C) Blockade of NMDAR prevented *astroSD*. CNO had no effect in Sham (D) or naive animals (E). (F) LFS-induced early-phase LTP was reversed by CNO. (G) D-AP5 prevented LTP reversal by CNO. (H) CNO paired with sciatic nerve stimulation (arrow; 100 mV, 50 Hz, 5 minutes, 0.1 milliseconds; *50 Hz + CNO*) induced *astroSP* in DREADD animals, which was prevented by D-AP5 (I). (J) 50-Hz low-intensity stimulation alone had no effect in DREADD animals. (K) *50 Hz + CNO* did not influence synaptic strength in Sham animals. Statistics: paired t-tests in (B-E) and (H-K); (F and G): One-way RM-ANOVA, *P < 0.05; for details, supplemental digital content (see Table S1, http://links.lww.com/PAIN/C383). Data are shown as mean ± SEM.

How can *astroSD* at spinal C-fiber synapses be reconciled with mechanical hypersensitivity in behaving animals, which, according to current knowledge, is typically associated with an amplification rather than a weakening of strength at spinal nociceptive synapses?^61^ We hypothesized that ascending–descending loops, which are inevitably distorted in spinal slice preparations, might contribute to this discrepancy.^69^ To assess the effect of astrocytic DREADD activation within intact neural networks, we recorded C-fiber–evoked field potentials in vivo in the SCDH of anesthetized rats^39^ (Fig. 3A). Consistent with findings from acute slice preparations, the intravenous (i.v.) administration of CNO (3 mg·kg^−1^) in the in vivo model depressed synaptic strength in 11 out of 13 animals, persisting throughout the 180-minute recording period (Fig. 3B). This suggests that *astroSD* occurs locally, independent of ascending or descending pathways.

Subsequently, akin to the in vitro experiments, we investigated the role of NMDARs in this phenomenon. Continuous perfusion of D-AP5 (50 µM) over the exposed SCDH prevented the induction of *astroSD* by CNO (Fig. 3C). Conversely, CNO had no effect on synaptic strength in Sham (Fig. 3D) or naive animals (Fig. 3E).

Manipulations that decrease synaptic strength may also reverse previously established synaptic strengthening under certain conditions.^76^ To test whether spinal astrocytes can achieve this, we administered CNO after electrical low-frequency stimulation (LFS) of C-fibers, which acutely amplifies synaptic strength at nociceptive synapses.^17^ This completely normalized synaptic strength (Fig. 3F). The astrocyte-mediated reversal of early-phase long-term potentiation (LTP), like *astroSD*, was prevented when NMDARs were blocked using D-AP5 (Fig. 3G).

Our in vitro experiments have shown that *astroSD* relies on postsynaptic calcium signaling (Fig. 1F). Earlier studies have demonstrated that the intensity, duration, and spatial pattern of neuronal calcium elevations play a pivotal role in determining the polarity of synaptic plasticity.^20,67^ We hypothesized that sustained low-threshold afferent input from movements and sensory interactions, which is absent in the in vivo model because of anesthesia-induced immobilization and is also missing in slice preparations, may be required, in combination with SCDH astrocyte activation, to sufficiently elevate postsynaptic calcium and promote synaptic potentiation at nociceptive synapses.

To test this idea, we stimulated the sciatic nerve at Aβ-fiber strength (50 Hz, 5 minutes, 100 mV, 0.1 ms) and paired it with an i.v. injection of CNO. And indeed, in contrast to CNO alone, the concomitant activation of peripheral low-threshold fibers^26,63,79^ and astrocytic GPCRs revealed a potentiation of strength at C-fiber synapses, persisting until the end of the recording period (Fig. 3H), suggesting that non-nociceptive afferent input can switch the polarity of astrocytogenic plasticity from depression to potentiation (acute astrocytogenic synaptic potentiation, *astroSP*). Notably, *astroSP* was also contingent on NMDAR activity, as it was blocked by D-AP5 (Fig. 3I). In contrast, 50-Hz stimulation alone had no effect on synaptic strength in deeply anesthetized animals (Fig. 3J). Similarly, the combination of afferent nerve fiber stimulation with CNO did not yield any effect on synaptic strength in Sham animals (Fig. 3K).

### 3.5. Microglia-dependent astrocytogenic synaptic potentiation might drive behavioral hypersensitivity to promote protective responses

During maladaptive pain associated with mechanical hypersensitivity, Aβ-fiber stimulation can increase excitation in the superficial SCDH via disinhibition of polysynaptic pathways.^73^ To test whether excitation can spread from deep to superficial layers in a similar manner when astrocytes are acutely activated by CNO, we next performed calcium imaging experiments.

**Figure 4.**
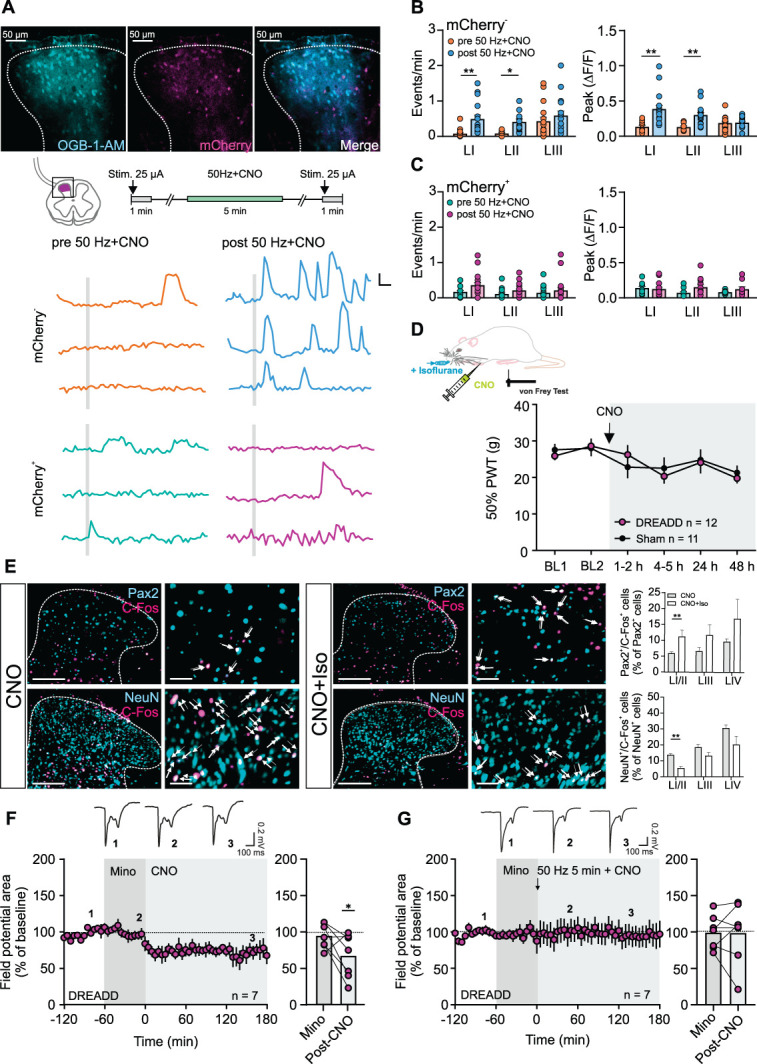
Stimulation of non-nociceptive fibers excites superficial SCDH neurons involving microglia after *50 Hz + CNO*. (A) mCherry^+^ slices were loaded with OGB-BAPTA-1-AM to assess calcium activity in mCherry^−^ and mCherry^+^ cells across SCDH laminae. Dorsal roots were stimulated at Aβ-fiber strength (25 µA) before (*pre-50 Hz + CNO*) and after (*post-50 Hz + CNO*) *50 Hz + CNO* application. Traces show recordings from lamina I cells (grey line: dorsal root stimulation, 25 µA; scale bar: 0.1 ΔF/F, 5 seconds). (B and C) *50 Hz + CNO* increased calcium event rate and response magnitude in mCherry^−^ cells (B) in laminae I/II, but had no effect in mCherry^+^ cells (C). (D) CNO administration under brief isoflurane anesthesia did not alter mechanical PWTs. (E) Representative images showing Pax2 or NeuN (cyan) and C-Fos (magenta) antibody staining in the SCDH. Scale bars are 200 µm and 50 µm, respectively. Arrows indicate colocalized cells. Quantification of colocalized cells is shown for different laminae. (F) Minocycline did not prevent *astroSD*, but fully blocked *astroSP* (G). Statistics: (B) One-Way ANOVA; *P < 0.05; **P < 0.01 (D) Two-Way RM ANOVA. (E) T-tests; **P < 0.01 (F and G) One-Way RM ANOVA; *P < 0.05. For details, supplemental digital content (see Table S1, http://links.lww.com/PAIN/C383). Data are shown as mean ± SEM.

Slices from DREADD animals were loaded with OGB-BAPTA-1-AM via patch pipettes (Fig. 4A). To test whether non-nociceptive afferent input affects calcium signaling in SCDH cells, we paired the bath application of CNO with an electrical stimulation to the dorsal root at Aβ-fiber intensity (25 µA, 50 Hz, 5 minutes, 0.1 ms; ie, *50 Hz + CNO*), as done in our electrophysiological experiments (Fig. 3H). We then monitored calcium activity in both mCherry^+^ (astrocytes) and mCherry^−^ (presumed neurons) cells across laminae in response to test stimuli at Aβ-fiber intensity (25 µA).

We observed calcium activity in mCherry^−^ cells in deeper laminae, whereas those in laminae I and II remained largely unresponsive to the test stimuli. However, after *50 Hz + CNO*, mCherry^−^ cells in laminae I and II displayed a significantly increased calcium activity with increased event rate and amplitude (Fig. 4B). Notably, this effect was absent when CNO was applied alone (see Fig. S6, supplemental digital content, http://links.lww.com/PAIN/C382). In contrast to mCherry^−^ cells, mCherry^+^ cells showed no change in response to the test stimuli after *50 Hz + CNO* (Fig. 4C; for details see Table S1, supplemental digital content, http://links.lww.com/PAIN/C383).

To assess the behavioral implications of our findings—specifically, whether simultaneous activation of Aβ-fibers and spinal astrocytes is required for the development of mechanical hypersensitivity—we administered CNO i.p. under deep isoflurane anesthesia, thereby suppressing afferent input during astrocyte activation. Von Frey testing was continued once animals had fully recovered from anesthesia. Notably, under these conditions, CNO had no detectable effect on mechanical thresholds (Fig. 4D).

Consistent with the behavioral findings, C-Fos expression in Pax2-negative (presumed excitatory) neurons of the SCDH was reduced in animals that were immobilized during CNO administration, whereas expression was increased in Pax2-positive inhibitory neurons, compared to animals receiving CNO without anesthesia or Sham controls (Figs. 4E and S7, supplemental digital content, http://links.lww.com/PAIN/C382). These results suggest that afferent input during astrocyte activation is required to engage the excitatory network and drive mechanical hypersensitivity.

We have recently demonstrated that the simultaneous activation of both microglia and astrocytes amplifies synaptic strength at SCDH C-fiber synapses.^39^ To explore microglia involvement in astrocytogenic plasticity, we pharmacologically inhibited microglia using minocycline (1 mM).^13,25^ Interestingly, this had no effect on the induction of *astroSD* (Fig. 4F). However, under these conditions, the induction of *astroSP* triggered by *50 Hz + CNO* was completely blocked (Fig. 4G), suggesting a context-dependent interplay between microglia and astrocytes in acute spinal plasticity.

Collectively, our findings support the hypothesis that astrocytes can transform touch into pain.^68^ By establishing a link between the deeper and superficial laminae in the SCDH involving microglia, astrocytes may facilitate interactions between these sensory pathways. In the context of acute pain and on a short timescale, this could be a protective adaptation to safeguard vulnerable tissues and promote healing.

## 4. Discussion

Using chemogenetic tools, in vivo and in vitro electrophysiological recordings, immunostaining, calcium imaging, and behavioral assays, we demonstrate that astrocytes mediate acute bidirectional plasticity at C-fiber synapses, thereby modulating nociception at the spinal level.

Under physiological conditions, astrocytes detect acute neuronal activity primarily via Gq-GPCRs on their plasma membrane,^37,57^ triggering intracellular calcium transients and the release of neuromodulatory molecules, including gliotransmitters.^22^ Here, we used Gq-DREADDs to selectively induce astrocytic calcium transients, mimicking their response to acute nociceptor activity.^38,64^ In acute slices as well as in intact, but deeply anesthetized animals, the chemogenetic activation of astrocytes using CNO triggered *astroSD*, which could represent a potential homeostatic response to temporarily elevated excitatory activity. Accordingly, the reversal of early-phase LTP at spinal nociceptive synapses upon astrocyte activation might contribute to restoring circuit homeostasis and reducing sensitivity to noxious stimuli under physiological conditions.

Astrocytes can enhance inhibitory synaptic transmission at cortical and hippocampal synapses.^6,34,46,49,74^ Consistently, CNO increased the rate of inhibitory currents in the SCDH, particularly those mediated by glycine receptors. Correspondingly, *astroSD* was glycine but not GABA_A_ receptor dependent. The underlying mechanisms driving this selectivity remain to be explored. *Glycine* plays a crucial role in nociceptive circuits,^19,21^ by directly or indirectly influencing the activity of lamina I projection neurons, thereby shaping spinal output.^14,24,50^ Although adenosine, generated from ATP released by spinal astrocytes,^9,79,82^ has been shown to enhance glycinergic transmission onto excitatory neurons via postsynaptic A1 receptors,^2^ this pathway appears unlikely to account for our findings. Specifically, we observed an increase in the rate, but not the amplitude of glycinergic IPSCs, and *astroSD* was independent of postsynaptic GPCR signalling. Although the precise source of glycine remains uncertain, the action potential–dependent increase in glycine release observed here suggests that it originated from glycinergic neurons rather than directly from astrocytes.^65^

*Glycine*'s dual role as a glycine receptor agonist and NMDAR coagonist may facilitate NMDAR-dependent mechanisms underlying *astroSD*^12,83^ as well as the reversal of early-phase LTP.^18^ Strikingly, the activation of astrocytic DREADDs had no effect on synaptic transmission in female rats. Although some evidence suggests that astrocyte contributions to nociceptive signaling may be sex-independent,^10^ our findings highlight a significant sex difference in astrocyte-mediated plasticity at the spinal level. The mechanisms underlying this sex-specific plasticity remain unclear and are the focus of ongoing investigations in our laboratory.

In awake, behaving animals, CNO administration increased mechanical sensitivity, likely because of sustained non-nociceptive afferent input associated with voluntary movement and interaction with the environment. Correspondingly, in vivo electrophysiological experiments showed that concurrent stimulation of low-threshold afferent fibers together with CNO (*50 Hz + CNO*) reliably induced NMDAR-dependent, astrocyte-driven synaptic potentiation (*astroSP*). This stands in contrast to a recent study^79^ reporting that Aβ-fiber stimulation alone suppressed synaptic strength at NK1R^+^ neuron synapses via astrocyte-mediated purinergic signaling and presynaptic inhibition through adenosine A1 receptors. Unlike that study, we used Gq-DREADDs to selectively and robustly activate astrocytic calcium signaling, producing immediate and widespread responses more akin to those observed after noxious stimulation of peripheral afferent fibers.^25,38^ Importantly, Gq-DREADDs activate GPCR-mediated signaling pathways that are distinct from purinergic mechanisms. Given the diverse functional roles of astrocytes, it is to be expected that stimulation through different molecular pathways may elicit markedly distinct physiological outcomes.

We propose that the strong astrocytic activation in our model, in combination with Aβ-fiber input, is sufficient to recruit microglia—likely via ATP or other astrocyte-derived signals—which may facilitate the propagation of Aβ-input into superficial dorsal horn circuits and promote *astroSP*. Supporting this, we found that although *astroSD* was microglia-independent, *astroSP* required microglial activation. Under these conditions, Aβ-fiber activity enhances, rather than suppresses, nociceptive signaling, likely via non-neuronal modulatory mechanisms.^39^ *AstroSP* may thus represent a cellular mechanism underlying acute mechanical hypersensitivity, potentially serving as an adaptive response to protect tissue during acute inflammatory or injury-related states.

Acute microglia activation has been demonstrated to rapidly reduce synaptic glycine receptors, but not GABA_A_ receptors,^8^ potentially unmasking polysynaptic excitatory circuits that relay low-threshold inputs to the superficial SCDH, thereby amplifying synaptic strength in output neurons.^71^ In line with this notion, we observed an increase in spontaneous calcium events with larger amplitudes in mCherry^−^ cells (presumed neurons) in the superficial SCDH in response to Aβ-fiber stimulation after *50 Hz + CNO*. Our study demonstrated that activation of inhibitory neurons, as indicated by C-Fos expression in Pax2^+^ cells, was markedly lower in the SCDH of animals receiving continuous low-threshold afferent input during CNO administration compared to those that did not. This suggests a diminished inhibitory tone under these conditions. Our data support the notion that astrocytes actively modulate inhibitory activity in the SCDH based on the network's state. Further studies are needed to elucidate the precise mechanisms by which astrocytes detect spinal network activity and regulate neuromodulatory release.

A previous study has demonstrated that targeted optogenetic activation of spinal astrocytes can induce both thermal and mechanical hypersensitivity.^52^ In contrast, we observed that CNO administration selectively increased mechanical sensitivity without affecting thermal thresholds. Although the mechanisms underlying this modality-specific effect remain unclear, similar findings have been reported after acute chemogenetic activation of a subset of spinal astrocytes expressing the transcription factor Hes5.^38,68^ These findings suggest not only that astrocytes modulate neuronal activity in an activation-dependent manner but also that distinct astrocyte–neuron–microglia signaling pathways may differentially regulate specific sensory modalities, both of which warrant further investigation.

In the present study, we chose a Gq-DREADD–based approach because it is well-suited to selectively mimic endogenous GPCR-mediated signaling in astrocytes^41^ and has potential for translational applications. We acknowledge that this is just one of many pathways that might be activated under both physiological and pathophysiological conditions, and the downstream effects may vary accordingly. Therefore, it will be exciting to explore whether spinal astrocytes exhibit stimulus-specific effects on transmission at nociceptive synapses.

Together, our findings highlight the critical modulatory role of astrocytes in acute nociception, demonstrating their ability to dictate the polarity of synaptic plasticity and fine-tune nociceptive responses based on concurrent environmental cues. Specifically, we show that astrocytic Gq-GPCR signaling, in the absence of simultaneous activity in low-threshold, non-nociceptive fibers, suppresses nociceptive transmission. Astrocytes respond to peripheral injury or tissue damage^33^ and can detect even brief activity in nociceptive fibers.^25,38^ By enhancing inhibitory tone and dampening nociceptive input, astrocytes may help prevent exaggerated responses to minor injuries, thereby maintaining pain perception within a physiological range. In contrast, when spinal astrocytes are activated alongside low-threshold afferents, they amplify synaptic strength at nociceptive synapses through microglial engagement, leading to mechanical hypersensitivity. Although typically associated with pathology, acutely increased transmission at SCDH nociceptive synapses—and the resulting mechanical hypersensitivity—may serve an important physiological function by heightening protective responses and supporting tissue recovery, eg, during acute neurogenic neuroinflammation.^77^ Evolutionarily, this adaptive mechanism likely promotes rest by diminishing non-noxious inputs such as light touch, pressure, and hair movement, thereby minimizing overall sensory input and reducing physical strain on the affected tissue.

In summary, depending on the state of the system, enhanced astrocyte activity can either reinforce or counterbalance acute nociceptive processing at the spinal level. This dynamic modulation effectively enhances the network's computational capacity. Although acute astrocyte activation was sufficient to reverse early-phase LTP in the anaesthetized animal, further research is needed to determine whether astrocytes can also reverse amplified nociception at later stages, potentially alleviating chronic pain. This could open new avenues for pain management in pathological conditions.

## Conflict of interest statement

The authors have no conflicts of interest to declare.

## Supplementary Material

## Appendix A. Supplemental digital content

Supplemental digital content associated with this article can be found online at http://links.lww.com/PAIN/C382 and http://links.lww.com/PAIN/C383.
