# Supplementary material for: Astrocytogenic bidirectional plasticity at spinal nociceptive synapses regulates acute nociceptive processing
Source: Pain. 2025 Sep 3;167(2):372–86. doi: 10.1097/j.pain.0000000000003805 (PMC12794346; doi:10.1097/j.pain.0000000000003805)
Supplement: Supplementary file 1 [file jop-167-372-s001.pdf]

Supplemental Digital Content

Astrocytogenic bidirectional plasticity at spinal nociceptive synapses regulates acute nociceptive processing

Sibel Ada, Laura Klinger, Hannah L. Teuchmann, Valeria Mussetto, Viktoria Hadschieff, Mira T. Kronschläger, Anna S.M. Siegert, Lidia Trofimova, Raphael Holzinger, Danijela Kurija and Ruth Drdla-Schutting

*Correspondence: ruth.drdla@meduniwien.ac.at

This PDF file includes:

Figures S1 to S7

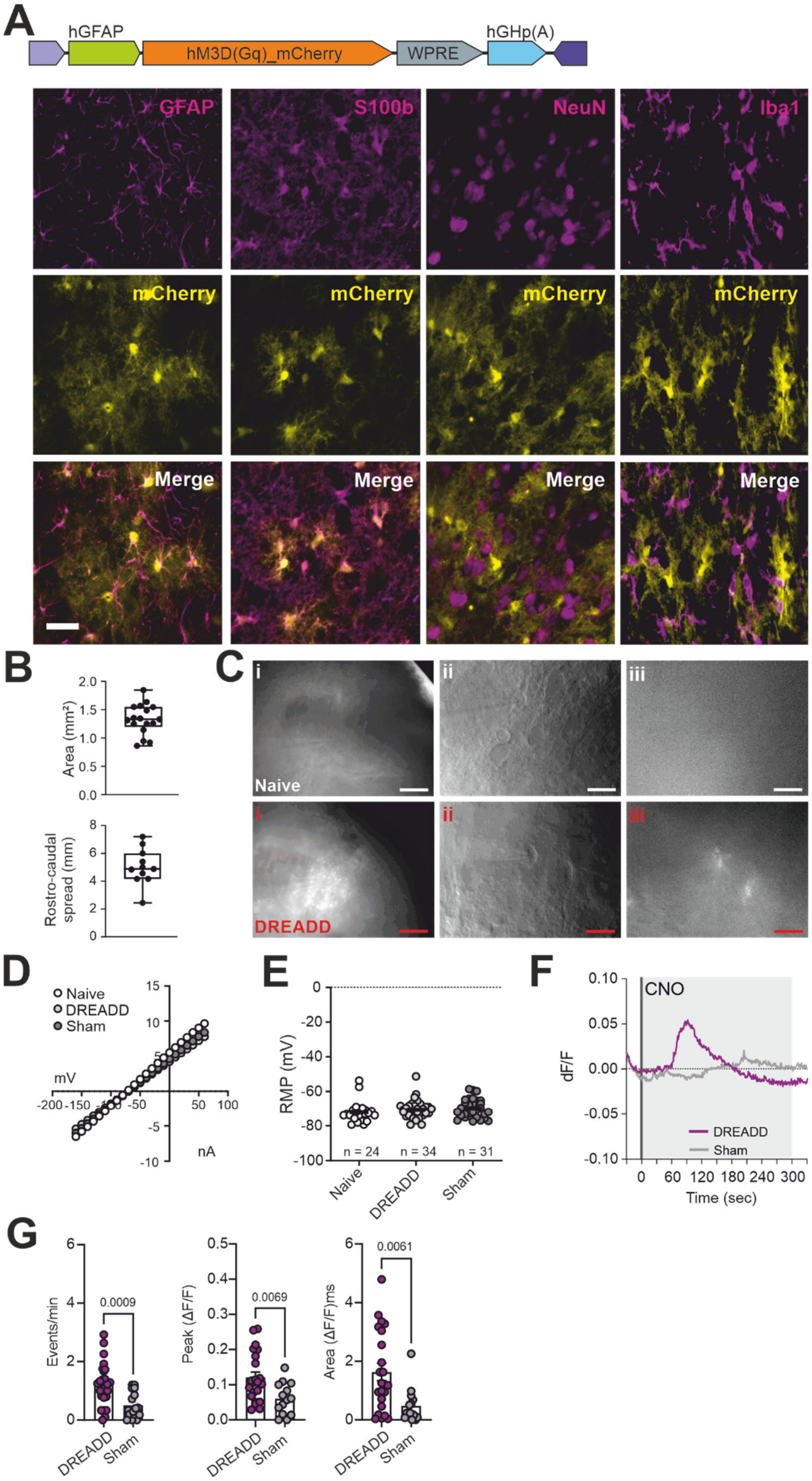
A
hGFAP
hM3D(Gq)_mCherry
WPRE
hGHp(A)
GFAP
S100b
NeuN
Iba1
mCherry
Merge
B
Area (mm²)
Rostro-caudal spread (mm)
C
i
ii
iii
Naive
DREADD
D
Naive
DREADD
Sham
mV
nA
E
RMP (mV)
n = 24
n = 34
n = 31
Naive
DREADD
Sham
F
CNO
dF/F
DREADD
Sham
Time (sec)
G
Events/min
0.0009
Peak (ΔF/F)
0.0069
Area (ΔF/F)ms
0.0061
DREADD
Sham

Figure S1: **DREADDs are specific to astrocytes in the SCDH, related to Figure 1.** (**A**) AAV-Gq-DREADDs, tagged with mCherry, were injected into the SCDH. mCherry+ cells were co-localized with astrocyte markers GFAP and S100β but did not overlap with the neuronal marker NeuN or the microglial marker Iba1. Scale bar = 50 µm. (**B**) Area of mCherry expression in 17 slices of 5 animals (in mm²) and rostro-caudal spread of mCherry expression in 11 animals (in mm). (**C**) Sample images of slices of naïve (upper panel) and DREADD injected animals (lower panel) are shown. **i**, Astrocytes were patched using glass pipettes. **ii**, Images are shown in higher magnification. **iii**, Fluorescence was used to visualize mCherry. Scale bar is 100 µm in **i**, 20 µm in **ii** and **iii**. (**D**) Current/voltage relationship for astrocytes in slices from naive animals (n = 24), and animals injected with AAV-Gq-DREADD (n = 34) or with the control virus (Sham, n = 31). (**E**) The resting membrane potential was comparable for astrocytes in all groups. Graphs show mean values ± SEM (**F**) CNO application induced an increase in intracellular calcium levels in astrocytes from DREADD-, but not from Sham animals. The graph displays the mean somatic calcium signal of mCherry-positive cells across 22 (DREADD) or 14 (Sham) slices. (**G**) DREADD activation was associated with an increase in the number of calcium events per minute, peak amplitude as well as area under the curve in astrocytes. Each dot represents the mean values of all detectable SCDH astrocytes per slice. T-Tests, for details see Table S1.

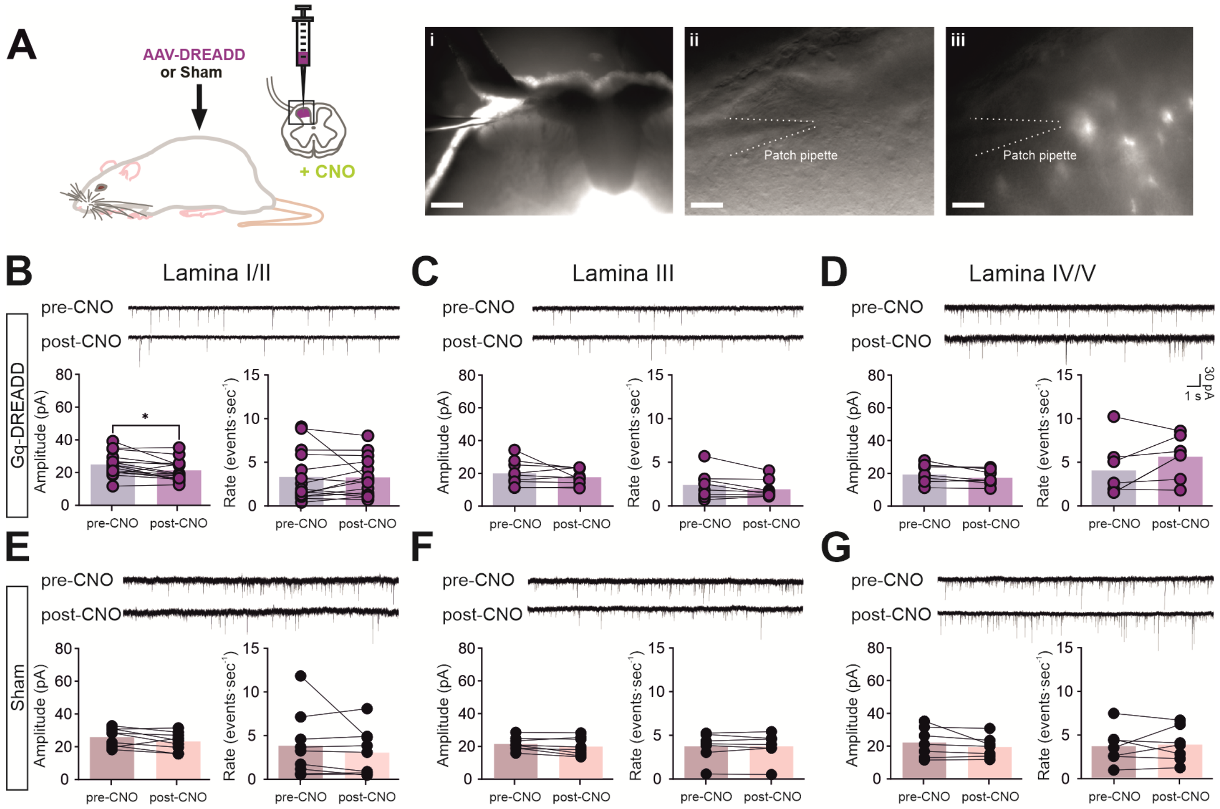

Figure S2: **The effect of CNO-mediated activation of astrocytic Gq-DREADDs on spontaneous EPSCs, related to Figure 1.** (**A**) Schematic of AAV-DREADD or Sham virus injection into the dorsal horn of the spinal cord. **i**, Representative image of a patched neuron in a spinal cord slice with dorsal root attached. **ii**, DIC image of the same slice at higher magnification. **iii**, Fluorescence image showing mCherry-expressing astrocytes in close vicinity to the patched neuron. Scale bars: 200 µm in **i**, and 25 µm in **ii** and **iii**. (**B**-**G**) sEPSCs were measured from neurons in different laminae of the spinal cord dorsal horn, as indicated. Graphs show the mean amplitudes and event rates of spontaneous EPSCs for time intervals before (-5-0 min, pre-CNO) and after (5-10 min, post-CNO) bath application of CNO. The traces display the original recordings from representative cells under the specified conditions. (**B**) CNO induced a reduction in sEPSC amplitude recorded in laminae I/II but did not affect amplitude or event rate in other laminae (LI/II: n = 15; LIII: n = 8; LIV/V: n = 7) (**C**, **D**). (**E-G**) CNO had no effect on sEPSCs in Sham animals (LI/II: n = 9; LIII: n = 7; LIV/V: n = 7). Data are shown as mean ± SEM. Statistics: Paired t-tests; * $P < 0.05$. Response values and statistics can be found in Table S1.

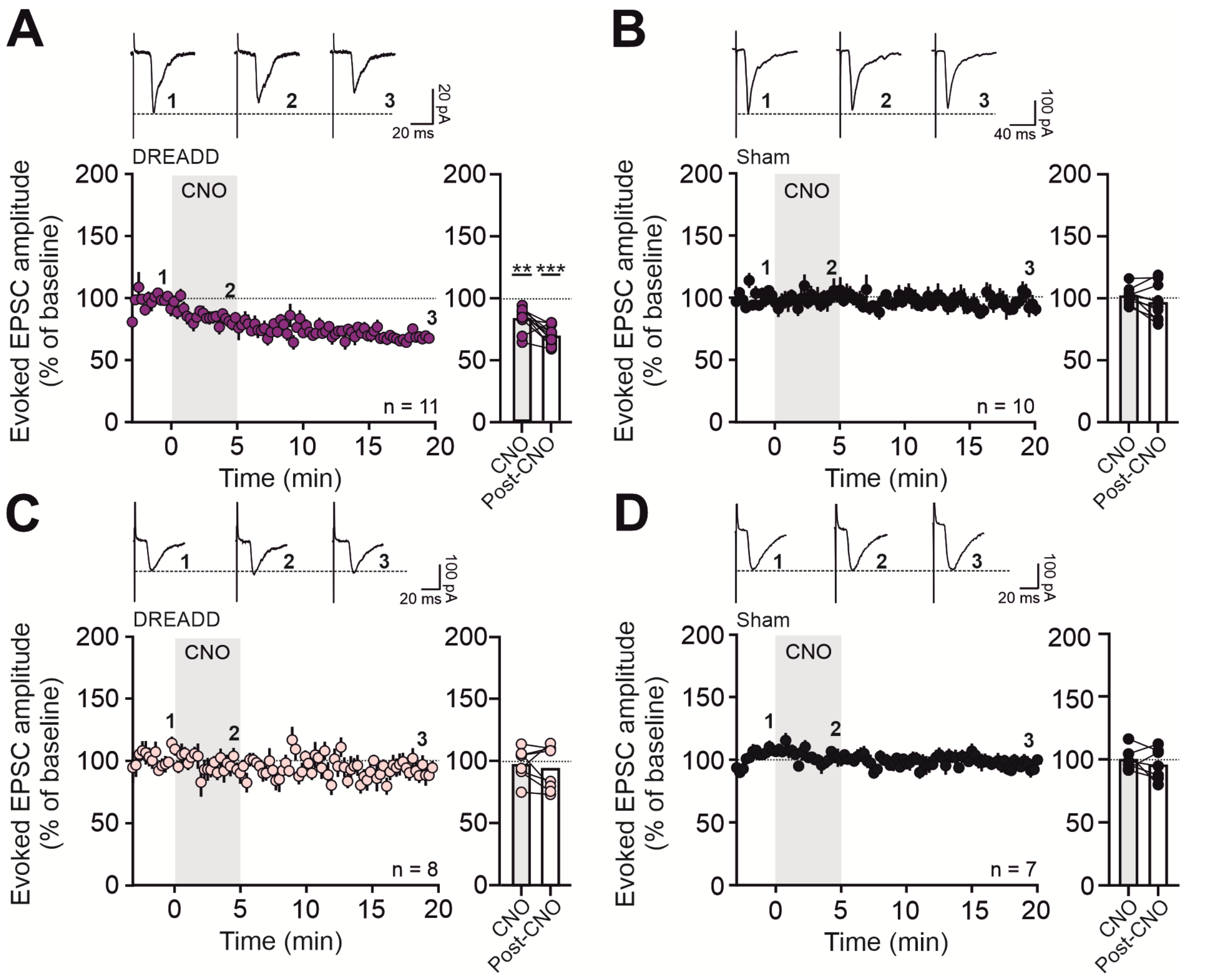

Figure S3: **Chemogenetic activation of SCDH astrocytes depresses strength at C-fiber synapses in males, but not in females, related to Figure 1.** In all graphs, the amplitudes of C-fiber-evoked EPSCs were analyzed, normalized to BL (dashed line), and plotted against time (minutes). Bar graphs show the BL-normalized mean values for each time period. CNO was bath-applied for five minutes from time point zero (grey area). Insets show example traces recorded at indicated time points. (**A**) DREADD activation induced robust *astroSD* in male DREADD-injected animals, but not in (**B**) Sham animals. (**C**) C-fiber-evoked EPSCs recorded in slices from females did not change upon astrocytic DREADD activation with CNO. (**D**) CNO was ineffective in female Sham animals; Statistics: One-way RM ANOVA; ** P = 0.0011; *** P < 0.0001. For details see Table S1; Data are shown as mean ± SEM.

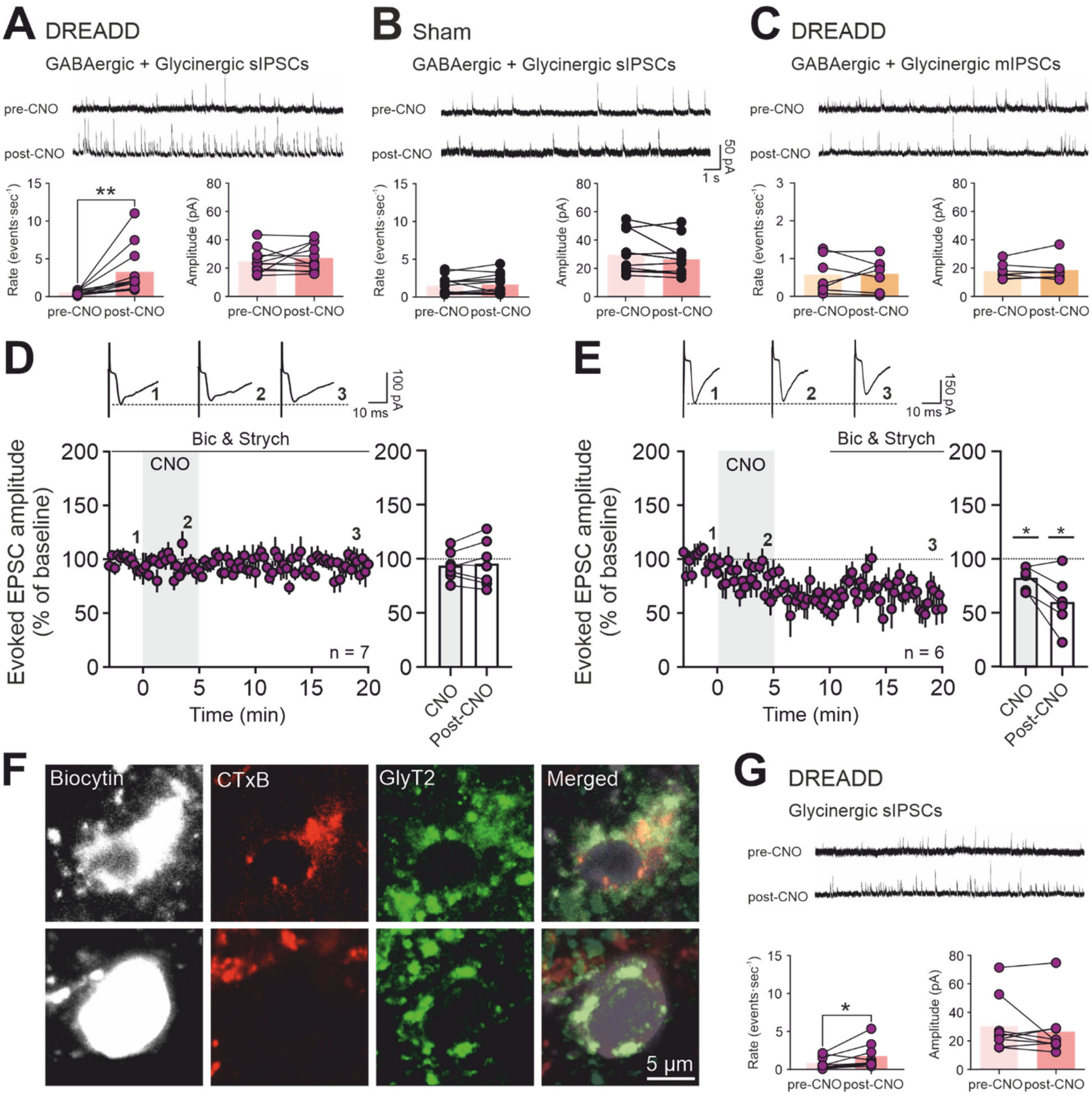

Figure S4: **AstroSD is glycine-receptor-dependent, related to Figure 1.** (**A**-**C**) Bar graphs show the mean amplitudes and event rates of sIPSCs and mIPSCs for time intervals recorded before (-5-0 min, pre-CNO) and after (5-10 min, post-CNO) bath application of CNO. Original traces show representative recording intervals for the indicated group. CNO increased the rate of sIPSCs in DREADD (n= 11) (**A**), but not in Sham animals (n = 11) (**B**), while amplitudes remained unaffected in both groups. (**C**) CNO did not affect rates or amplitudes of mIPSCs (n = 7). (**D**, **E**) In both graphs, the amplitudes of C-fiber-evoked EPSCs were analyzed, normalized to BL (dashed line), and plotted against time (minutes). Bar graphs show the BL-normalized mean values for each time period. CNO was bath-applied for five minutes from time point zero (grey area). Insets show example traces recorded at indicated time points. Black lines indicate the period of substance application. (**D**) The bath application of bicuculline and strychnine fully prevented the induction of *astroSD*. (**E**) Blocking $GABA_A$ and glycine-receptors after the wash-out of CNO had no effect on the maintenance of *astroSD*. (**F**) Immunostaining of biocytin-filled spino-LPBN neurons ($CTxB^+$, red) as well as unidentified neurons with C-fiber input showed dense glycinergic innervation (GlyT2, green). (**G**) Graph shows event rates and amplitudes of pharmacologically isolated glycinergic sIPSCs. CNO induced

an increase in the event rate, while the amplitude remained unchanged (n = 9). Statistics: (**A**-**C**, **G**) paired t-tests; * $P < 0.05$; (**D**, **E**) One-way RM ANOVA, * $P < 0.05$; ** $P < 0.001$. For details see Table S1; Data are shown as mean ± SEM.

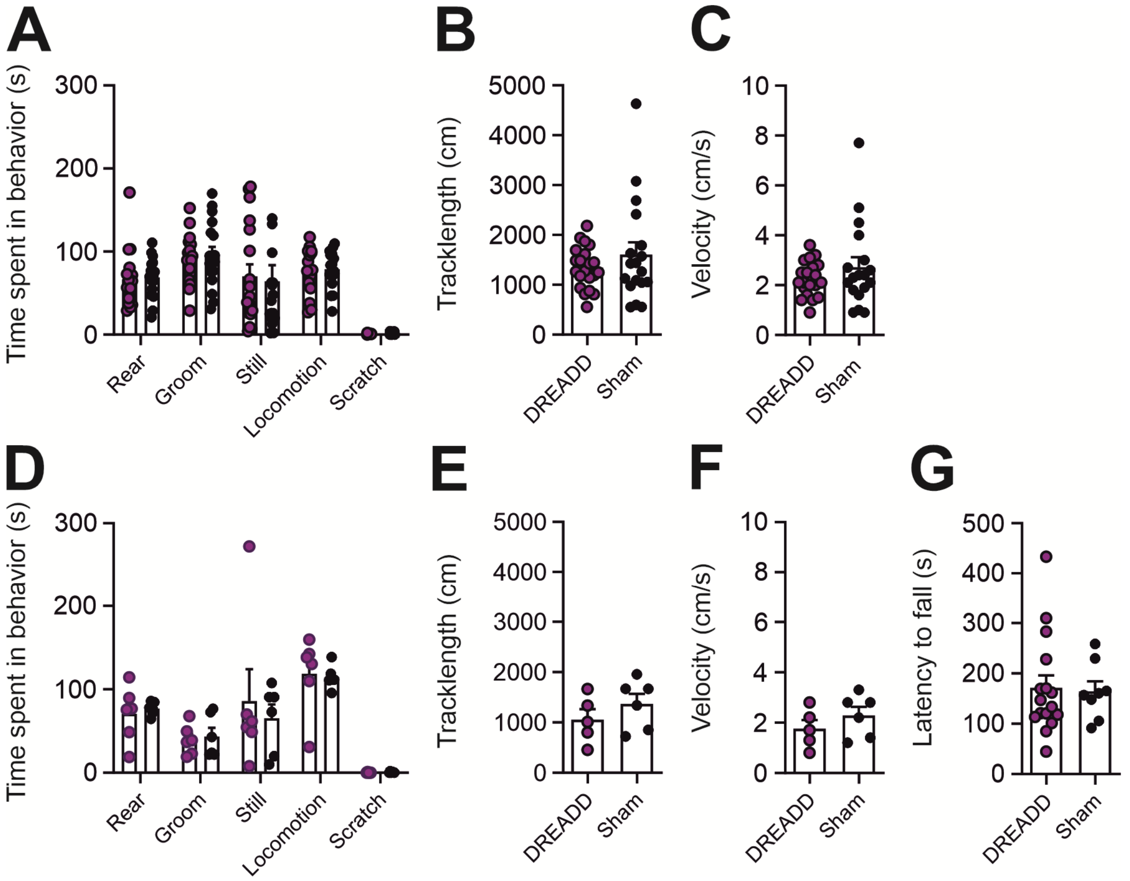

Figure S5: **CNO had no effect on spontaneous behaviors and motor performance, related to Figure 2.** All animals received an i.p. injection of CNO. At the testing time point (10 min post injection in **A**-**C**; DREADD n = 21, Sham n = 18; or 90 min post injection in **D**-**F**; DREADD n = 6, Sham n = 6), all animals were subjected to the spectroscopy apparatus for 10 min. The graphs show the automated classification of voluntary behaviors. DREADD animals are shown in magenta, Sham animals in black. (**G**) shows the latency to fall in seconds in the rotarod test (DREADD n = 16, Sham n = 8). Response values and statistics can be found in Table S1.

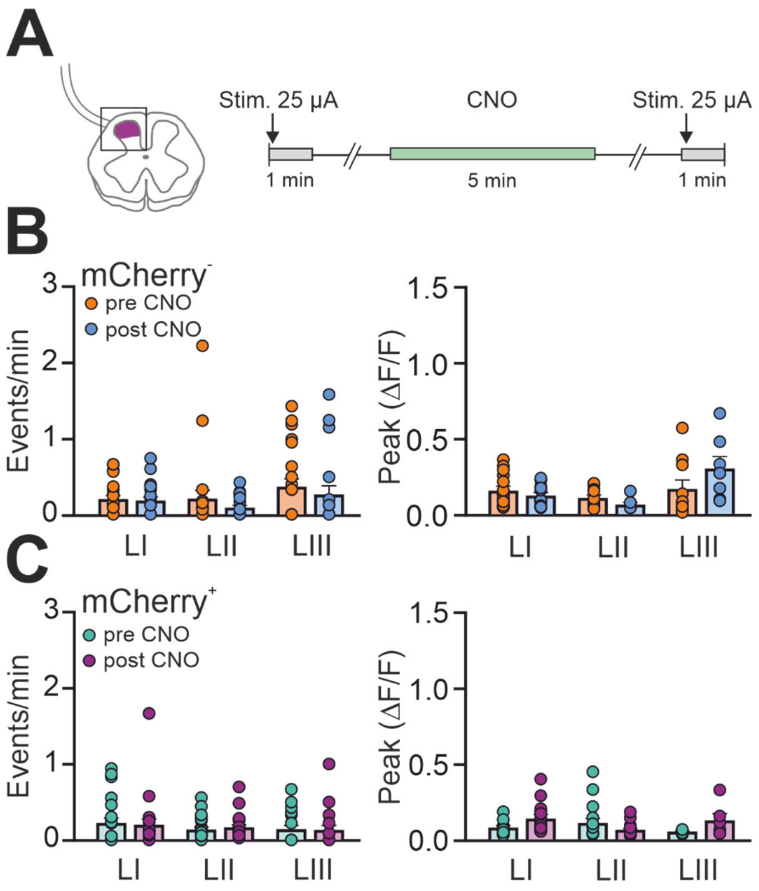

Figure S6: **Calcium activity in response to electrical stimulation of the dorsal root at Aβ-fiber strength does not change following the application of CNO, related to Figure 4.** (**A**) Calcium activity in response to electrical stimulation of the dorsal root at Aβ-fiber strength was assessed in (**B**) mCherry$^-$ and (**C**) mCherry$^+$ cells in the spinal cord dorsal horn before (pre-CNO) and after (post-CNO) bath application of CNO for 5 minutes. No changes were detected in the rate or amplitude of calcium events in either mCherry$^-$ or mCherry$^+$ cells. Response values and statistics can be found in Table S1.

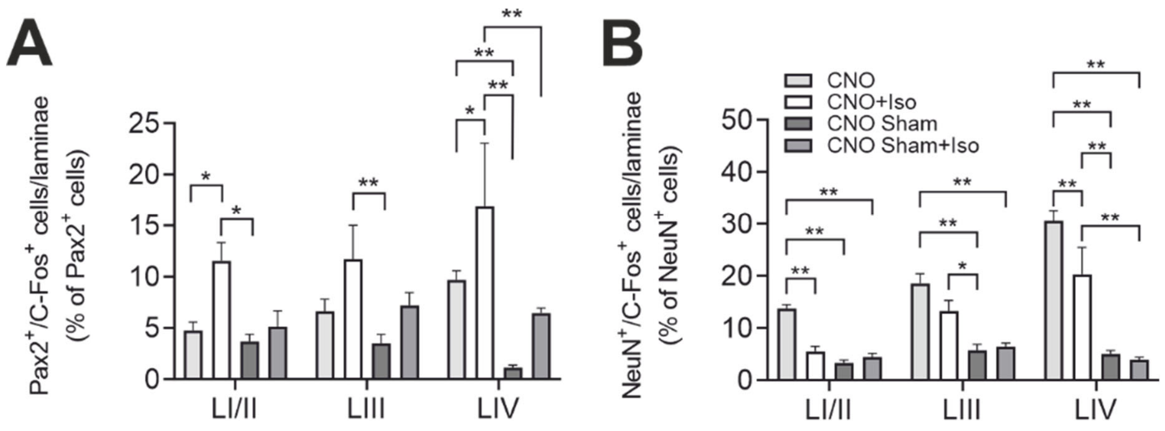

Figure S7: **Immobilization with isoflurane during CNO administration enhances C-Fos expression in Pax2-expressing cells in the SCDH, related to Figure 4.** Quantification of co-localized (**A**) $Pax2^{+}$/$C\text{-}Fos^{+}$ and (**B**) $NeuN^{+}$/$C\text{-}Fos^{+}$ cells across different laminae of the SCDH is shown for both DREADD- and Sham animals. Statistics: Two-Way ANOVA; * $P < 0.05$; ** $P < 0.01$; for details, see Table S1. Data are shown as mean ± SEM.
